# Associations of sugar-related food parenting practices and parental feeding styles with prospective dietary behavior of children and adolescents: a systematic review of the literature from 2017 to 2023

**DOI:** 10.3389/fpubh.2024.1382437

**Published:** 2024-08-14

**Authors:** Hannah Leonike Hübner, Tina Bartelmeß

**Affiliations:** Faculty of Life Sciences: Food, Nutrition and Health, University of Bayreuth, Kulmbach, Germany

**Keywords:** sugar-rich foods and beverages, children, food parenting practices, adolescents, long-term associations, parental feeding styles

## Abstract

**Introduction:**

High consumption of sugar-rich foods and beverages has been associated with increased overweight and obesity in children and adolescents. Dietary behavior is influenced by learned mechanisms that originate in childhood and is associated with food parenting practices (FPP) and parental feeding styles (PFS). This systematic review aimed to narratively synthesize FPP and PFS concerning sugar-rich foods and beverages and their associations with the prospective dietary behavior of children and adolescents to derive evidence-based recommendations for health professionals and parents to promote healthy behaviors.

**Methods:**

A systematic literature search was conducted using PubMed and Web of Science databases covering the publication years 2017–2023. The results were narratively synthesized, and exposure-outcome matrices were used for visual representation. The review included 15 peer-reviewed studies from different geographical regions that investigated FPP or PFS regarding the handling of sugar-rich foods and beverages in children’s diets and reported the associations with the prospective dietary behavior of children and adolescents.

**Results:**

The findings indicate that highly controlling parental practices were associated with the development of unhealthy eating behaviors and preferences for sugar-rich foods and beverages over time. Conversely, parental practices that emphasized structure and balance in dietary choices yielded more positive long-term outcomes, associated with reduced preferences for sugar-rich foods and drinks.

**Discussion:**

The results underscore the significance of fostering a healthy home environment and active parental role modeling in promoting healthier dietary behaviors among children and adolescents.

## 1 Introduction

The consumption of sugar-rich foods and beverages during childhood has been identified as a significant determinant in the development of overweight and obesity, along with associated comorbidities, and non-communicable diseases, such as type 2 diabetes, fatty liver disease and dental caries (1–3). This, in turn, places a substantial financial strain on healthcare systems over the long term (4). In some nations, particularly the United States, childhood overweight and obesity have reached epidemic levels (5). Furthermore, the prevalence of overweight and obesity among children and adolescents remains high in European countries with overall 29% of children aged 7–9 years across 33 European countries affected by overweight, including cases of obesity (6). To prevent health complications associated with high consumption of sugar-rich foods and beverages, the World Health Organization (WHO) recommends limiting the intake of free sugars to less than 10 % of total daily energy intake for both adults and children, with an ideal target of less than 5% (3). Free sugars are sugars that are added to food and beverages. However, free sugars also occur naturally in honey, syrup, fruit juice concentrates, and fruit juices. Free sugars contribute to the total energy density of the diet and can trigger a positive energy balance that promotes the development of overweight and obesity (3). Limiting the consumption of energy-dense foods rich in free sugars, such as sweets, cakes, biscuits, chocolate, sodas, and juice drinks, by children and adolescents is a growing concern for health organizations, policymakers, and parents (7, 8). Hence, understanding the factors contributing to high sugar consumption is of paramount importance for effective public health management and public and private prevention strategies.

Parenting plays a significant role in shaping children’s dietary habits from early childhood through adolescence (9–12) and can contribute to health-promoting or health-adverse behaviors (13). Recent studies have identified a relatively new food parenting style, termed “overprotection,” which is considered significant for children’s eating behaviors and reflects parental concerns about their impact on their children’s dietary habits. Overprotective parents reported using parenting practices that are known to be positively associated with children’s food intake, such as modeling healthy eating behaviors, but also less favorable practices, such as applying pressure. However, longitudinal data on parental practices and their associations with children’s healthy eating are lacking. Such data are needed to enhance communication and interventions for parents to improve their children’s food intake, reinforce important dietary strategies positively affecting children’s eating behaviors, and address parenting styles with unintended negative long-term consequences (14). Therefore, understanding how parental practices concerning the handling of sugar-rich foods and beverages are associated with children’s behavior is essential. However, there is a lack of an overview of the links between different food-related parenting practices with a focus on the approach to sugar-rich foods and beverages and the associations with the prospective dietary behavior of children and adolescents. Therefore, this systematic review aims to address this gap by (a) systematically collecting evidence from longitudinal studies examining sugar-related food parenting practices and the long-term associations with children’s behavior, (b) summarizing the existing evidence narratively to provide an overview of the associations between food parenting around sugar-rich foods and beverages and children’s prospective behavior, and (c) deriving recommendations for health professionals and parents on sugar management in food parenting practices.

## 2 Food parenting practices, parental feeding styles, and children’s dietary behavior

The concept of “food parenting” includes food-related parenting practices (FPP) and parental feeding styles (PFS) (13, 15). FPP refer to parental behaviors that influence a child’s food-related attitudes and are divided into three larger domains, each with specific subcategories (15, 16): coercive control, structure, and autonomy promotion (for an overview, see Supplementary Figure S1). Coercive control practices describe parental attempts to dominate, pressure or impose their will on the child’s eating behavior. This includes practices such as restrictions, pressure to eat, or the use of threats and bribes (15). The FPP domain of structure describes the organization of the child’s environment by parents to promote the child’s nutritional literacy. This includes practices such as monitoring, modeling, rules and limits, and the availability of food (15). Autonomy support aims to support psychological autonomy and promote independence. It includes approaches such as nutritional education, encouragement or child involvement (15). To further differentiate FPP in terms of children’s awareness, Ogden et al. (17) devised categories of “overt” and “covert” control. Overt control practices involve parents deciding what, when, where, and how much children should eat, while covert control practices, such as providing healthy food options or avoiding restaurants with unhealthy food, are not recognized by the child. In general, coercive control practices are associated with a negative impact on children’s dietary behavior and weight development, whereas structural and autonomy-enhancing parenting practices are associated with positive impacts on children’s dietary behavior and weight development (15).

Parental feeding styles describe the more general interactions between parents and their children across food-related situations. Based on Baumrind’s (18) taxonomy of general parenting styles, the PFS are defined by the two key dimensions demandingness (the degree of control parents exert), and responsiveness (warmth and acceptance in response to their children’s needs) (13, 19). Within this framework, four distinct PFS are outlined. Authoritative parenting is characterized by a high level of demandingness and clear rules, coupled with a high level of responsiveness to children’s needs. Authoritarian parenting is marked by high demandingness and rules but with lower responsiveness and less consideration of the child’s needs. Indulgent parenting features low demandingness and high responsiveness, with few strict rules, but substantial engagement with the child’s needs. Finally, uninvolved parenting is associated with low demandingness and responsiveness (for an overview, see Supplementary Figure S2) (13, 19). In general, indulgent and uninvolved feeding styles are associated with an increased body mass index (BMI) (20), while an authoritative style, in particular, tends to yield the most favorable outcomes for children and is associated with a healthy BMI (13). Overall, non-responsive, controlling practices tend to be associated with negative health outcomes, while responsive practices, which are characterized by developmentally appropriate responses toward the child, support health-promoting behaviors (21).

## 3 Methods

The Preferred Reporting Items for Systematic Reviews and Meta-Analyses (PRISMA) 2020 statement by Page et al. (22) was used to guide the research process. The literature search for the systematic review was conducted in two databases—Web of Science and PubMed—in September 2023. A search strategy was developed based on the research question, and the retrieved results were selected using inclusion and exclusion criteria through multiple process steps. Subsequently, a manual search was performed to include potentially relevant literature that may have used different terminologies.

To generate an appropriate search strategy, key terms were derived based on the research question and the selection criteria were formed, utilizing various combinations and synonyms. The search consisted of three core elements: “parenting practice,” “sugar,” and “behavior.” Additional terms included for example “parenting style,” “parenting strategy,” “unhealthy,” “energy-dense,” “preference,” and “consumption.” Following the example of Shloim et al. (13), the terms “children” or “adolescents” were omitted in the strategy to avoid excluding studies that used unconventional descriptions such as “eighth graders” or “preschoolers.” The final search strategies for each database are listed in Supplementary Table S1.

### 3.1 Inclusion and exclusion criteria

Inclusion and exclusion criteria were defined based on the modified PEO framework proposed by Khan et al. (23). The components consisted of specified population (P), exposure (E), and outcome (O). This study expanded the PEO framework by including Publication Type (PT).

As population (P), children and adolescents aged 6 months to 16 years were of interest. The choice of this age range was based on the recognition that the concept of food-related parenting can be applied from 6 months of age onwards (24). From the age of 16, it can be expected that external influences, such as peer groups or increasing autonomy and freedom of movement, significantly affect children’s behavior (9). Therefore, conclusions about parenting practices and feeding styles from this age onward are highly inaccurate. When selecting the studies, this time frame included both the age of the subjects at the first assessment and the age at which the outcomes were measured in the longitudinal studies. In addition, the participants in the studies had to be generally healthy (without physical, physiological or psychological limitations that could bias the results).

As exposure (E), the FPP and PFS concerning the handling of sugar-rich foods and beverages in children’s diets were of interest. In the studies, parental management of sugar could have been addressed in terms of describing measures such as prohibiting or authorizing the consumption of sugar-rich foods and beverages, setting rules or indirectly by avoiding such foods and drinks in the child’s environment, or directly as specific FPPs or PFSs. Studies focusing on parenting related to physical activity, sleep, media, or similar topics, as well as those examining early feeding practices in the first 6 months of life, were excluded. In addition, this review included studies that focused on or at least explicitly considered foods and beverages in FPP and PFS, which may contain large amounts of free sugars. In the studies reviewed, these foods and beverages are not always labeled as containing free sugars or as sugar-rich, which is why terms such as “unhealthy,” “energy dense,” and “snacks” or “sweets” were also used to describe them in the studies. This review included studies that referred to these food descriptions and dietary patterns in their research designs or reports, suggesting that parental handling of sugar-rich foods was also investigated or included in the analysis.

The outcome (O) of interest was the prospective behavior of children and adolescents. This encompassed not only dietary behavior but also social and cognitive aspects. Additionally, the measurement outcomes indicating specific dietary behaviors (behavioral indicators), such as BMI or body fat mass, were included. For a study to be included in the review, a prospective, retrospective, or theoretical association between the previously exposed FPP or PFS and the prospective behavior or behavioral indicators of the children had to be demonstrated, with a minimum time interval of 6 months between the exposure and behavioral assessment. Cross-sectional studies as well as experimental studies that assessed dietary behavior immediately following a short-term stimulus were excluded from the analysis, as they do not represent the prospective effects of FPP and PFS as well as parenting in the natural and home environments.

Finally, the document type was limited to “Articles,” and the publication date was restricted to the years “2017–2023.” By narrowing the timeframe, the timeliness of the findings can be ensured. No specific criteria were set within the methods, allowing for the inclusion of both quantitative and qualitative research as well as mixed methods approaches.

### 3.2 Selection of studies

**Figure 1:**
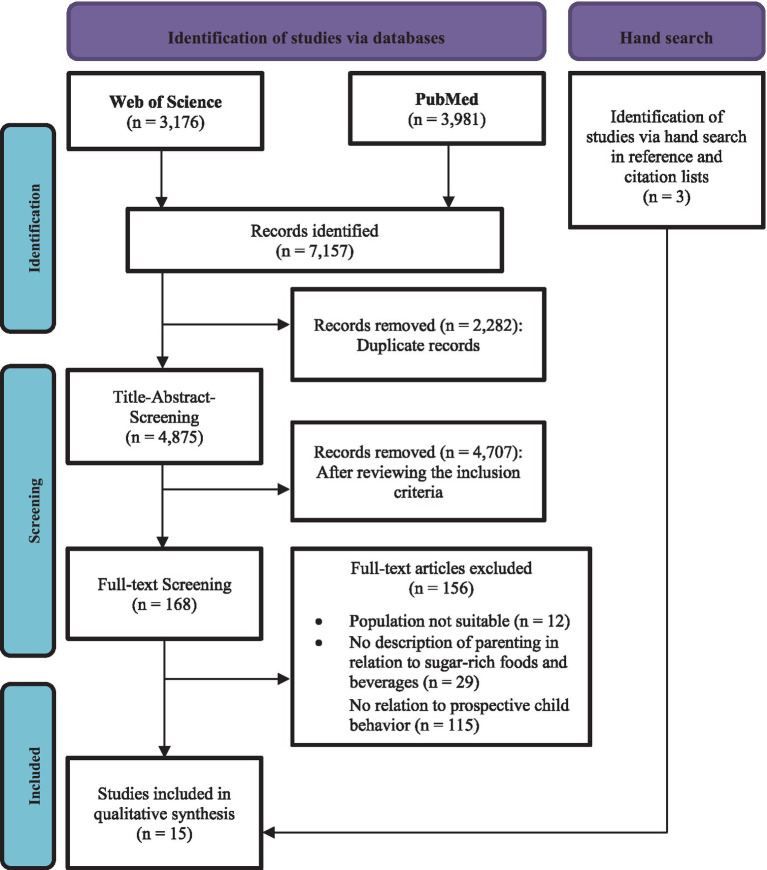
PRISMA Flow diagram.

The search in PubMed yielded 3,981 hits, and an additional 3,176 hits were obtained from Web of Science. Thus, a total of 7,157 articles were identified using the search strategies (see Supplementary Table S1). Duplicates (*n* = 2,282) were removed, leaving 4,875 studies. Subsequently, the relevance of these studies was assessed based on their titles by searching for relevant keywords to exclude studies that did not relate to the population. If the initial selection was positive, the abstract of the study was examined for the inclusion and exclusion criteria. Consequently, full-text screening was conducted (*n* = 168). For articles that did not meet at least one of the inclusion criteria, the primary reason for exclusion was recorded (*n* = 156). Through this process, 12 studies were identified for narrative synthesis. Three additional studies were identified through a manual search of the reference lists (see PRISMA-Flow diagram in Figure 1).

### 3.3 Data analysis and synthesis

The included studies (*n* = 15) were imported into the MAXQDA 2022 data analysis software for narrative synthesis. A narrative synthesis uses a textual approach to analyze the relationships within and between studies, offering a comprehensive assessment of the robustness of the evidence concerning the phenomena of interest (25). The selected studies were assessed by the authors using the Joanna Briggs Institute (JBI) checklists for cohort studies and randomized controlled trials (26). An overall quality assessment was carried out to classify the studies as “positive,” “moderate,” or “negative,” which yielded no studies having to be excluded from the analysis.

To extract and narratively synthesize the data, a content analysis was carried out according to the principles of Kuckartz and Rädiker (27). Categories were formed *a priori* (deductive) and supplemented with inductive categories throughout the process. The deductive categories encompassed bibliometric data, study design, methodology (e.g., type and frequency of data collection), and exposures (FPP and PFS). The categories for the study outcomes (e.g., behavioral outcomes) were compiled based on the tripartite categorization of Stok et al. (28) (“food intake,” “food choice,” and “eating behavior”) and assigned to the corresponding exposure. This distinction makes it possible to illustrate and demonstrate the associations of different parental practices with children’s behavior in a more nuanced way. A differentiation is drawn between (1) food choice, which includes outcomes that precede the actual consumption of food (e.g., preferences, tastes, and intentions); (2) eating behavior, which includes outcomes that are associated with the actual act of eating (e.g., frequency, quantity, habits, and diets); and (3) food intake/nutrition, which includes all outcomes related to what is consumed (e.g., healthy versus unhealthy food intake, dietary patterns, and food components) (28). As health-related behavioral indicators were also included, a fourth inductively formed category was labeled “indicators of health behavior.” The analysis was conducted based on these categories, along with a visual representation in the form of a tabular overview (exposure-outcome matrix). The matrix presentation aims to provide a quick overview of relevant results. The results related to the prospective behavior of children and adolescents are represented by arrows pointing upwards or downwards, indicating a decrease or increase in the corresponding behavior. To quickly grasp the nature of the associations, the symbols are additionally colored red (negative behavior) or green (positive behavior). Non-significant associations are denoted by the black dots. Associations that yielded significant associations in the opposite direction (behavior as a predictor of parenting) are represented by an inverted “A” symbol.

## 4 Results

### 4.1 Description of included studies

The systematic literature search yielded a total of 15 studies that met the inclusion criteria (see Supplementary Table S2). While there are a large number of studies and also systematic reviews addressing parent–child interactions in relation to diet (29–32), few explicitly consider sugar-related practices and the prospective associations with children’s and adolescents’ behavior, which is the focus of this review.

Among the included studies, there were 14 prospective longitudinal studies and one randomized controlled trial (RCT). Most studies were conducted in Europe (*n* = 9), followed by Australia (*n* = 4), North America (*n* = 1), and Asia (*n* = 1). The time span between the baseline assessment and first follow-up ranged from 10 months to 6 years. Although no restrictions were placed on methodology, no qualitative studies were identified. This can be attributed to the frequent use of questionnaires in this research area to assess FPP or PFS, such as the Feeding Practices and Structure Questionnaire (FPSQ) (33). The most commonly used questionnaires to elicit FPP or PFS was the Child Feeding Questionnaire (CFQ) by Birch et al. (34) and the Comprehensive Feeding Practice Questionnaire (CFPQ) by Musher-Eizenman and Holub (35). Some studies used selected items of recognized scales or formulated their own questions and response options to collect data to infer a particular FPP or PFS. In one case, parenting practices were assessed through observation rather than questionnaires (36). The measurements of children’s prospective behavior varied greatly, ranging from questionnaires to face-to-face interviews, experimental assessments, and anthropometric measurements.

### 4.2 Synthesis of results

Of the 15 studies included in this review, eight different FPP and one PFS related to sugar-rich foods and beverages were examined. Some studies have investigated multiple practices concurrently, resulting in 35 data sets on prospective associations with children’s and adolescents’ behavior that will be narratively synthesized in this chapter using exposure-outcome matrices.

The most frequently studied type of FPP was restrictive feeding (*n* = 9), followed by the use of food as a soothing strategy (*n* = 5), food availability (*n* = 5), permissiveness and control of food choices (*n* = 4), and use of food as a reward (*n* = 4). Less frequently, studies focused on parenting practices such as modeling (*n* = 3), monitoring (*n* = 3), nutrition education (*n* = 1), and the promotion of balance and variety in diet (*n* = 1). Practices and styles assessed under synonymous terms are included in the terms listed above. To ensure consistent terminology and to make the results more comparable, the parenting practices were subsequently categorized into “coercive control,” “structure,” and “autonomy support” based on the basic structure proposed by Vaughn et al. (15) and the revision by Musher-Eizenman et al. (16) (see Supplementary Figure S1). However, unstructured practices, involving high permissiveness or neglect regarding nutrition were categorized separately under the label “permissiveness” and handled as a PFS. Furthermore, the results of the dietary behavior were divided into three dimensions of food intake, food choice, and eating behavior according to Stok et al. (28). As described above, an additional dimension was added for indicators of health behavior.

### 4.3 Associations with the FPP coercive control

The FPP of coercive control includes parenting practices of restriction, using food to soothe and using food as a reward.

#### 4.3.1 Restriction

The restriction of sugar-rich and other energy-dense foods, in general, was examined in nine longitudinal studies (36–44). Various approaches and multiple behavioral outcomes were investigated regarding restriction (see Table 1). The age of the children during the study period ranged from 3 to 11 years, with a follow-up period of 10 months to 6 years and three assessment points. Among the nine included data sets, 13 behavioral outcomes from all three domains of dietary behavior were investigated.

**Table 1:** Exposure-outcome matrix of the associations between restriction and prospective behaviors.

FPP	Prospective child behavior	Prospective longitudinal studies								
		Barbosa et al. (44)	Boots et al. (42)	Boots et al. (37)	Derks et al. (38)	Derks et al. (43)	Farrow et al. (36)	Haszard et al. (39)	Liszewska et al. (40)	Toh et al. (41)
High level of restriction	Food intake									
	Consumption									
	Energy-dense foods	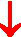								
	Nutrient-dense foods	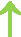								
	Snack consumption									
	Unhealthy snacks		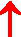							
	Healthy snacks		•							
	Food choice									
	Preference for:									
	Sweets			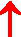						
	Fruit & Vegetables			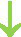						
	Eating behavior									
	Food fussiness									
	Enjoyment of food					•				
	Emotional overeating					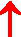				
	Food responsiveness					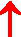				
	Satiety responsiveness					•				
	Food reward									
	Health behavior indicator									
	Body fat mass				•, ∀					
	BMI			•	•, ∀		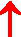	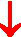	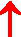 , ∀	

SR, Systematic review; colors: green: healthy behavior, red: unhealthy behavior; 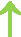/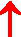: increased healthy/unhealthy behavior; 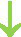/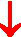: decreased healthy/unhealthy behavior; •: no significant association; ∀: association in the opposite direction (behavior as a predictor of increasing/decreasing use of parenting practice).

Food intake, assessed by dietary patterns of energy-dense or nutrient-dense foods and unhealthy (sugar-rich) and healthy (low in free sugars) snacks, was examined in two studies (42, 44). These studies have yielded ambivalent results. While one study found that girls whose parents used more restriction at 4 years of age consumed less energy-dense and more nutrient-dense food at age 7 (44), another study found that the association was opposite in terms of snack consumption (42). Boots et al. (42) found a significant positive association between restrictive feeding and later intake of unhealthy snacks but no significant association with the intake of healthy snacks. Food choice, as reflected in the preferences for sweets, fruits, and vegetables, was explored by Boots et al. (37). The authors demonstrated that restriction significantly increased the preference for sugar-rich sweets and decreased the preference for fruits and vegetables. Two studies reported significant results regarding children’s prospective eating behavior. Derks et al. (43) examined the impact of restriction on the enjoyment of food but did not find significant associations. They further investigated emotional overeating and responsiveness to food, both of which were positively associated with exposure. Additionally, Toh et al. (41) demonstrated an increase in reward responsiveness to food as a long-term response to sugar-rich food restrictions. Furthermore, five studies examined the associations of restrictive parenting practices with indicators of health behavior, such as body fat mass and BMI. While Boots et al. (37) and Derks et al. (38) found no significant associations with BMI or reported an inverse association; Farrow et al. (36) and Liszewska et al. (40) observed a significant increase, whereas Haszard et al. (39) observed a decrease. Additionally, Liszewska et al. (40) also revealed a reverse association, indicating that a higher BMI leads to subsequent use of restriction. Derks et al. (38) reported significant associations between fat mass and subsequent restriction; however, these associations were not in the originally hypothesized direction. It can be concluded that in eight cases, the use of restriction was found to be associated with unhealthy behaviors including high consumption of foods and beverages rich in free sugars, whereas in one study, it was associated with a decrease in healthy behaviors (37). Only two studies have indicated a reduction in unhealthy behaviors due to restrictive food parenting (39, 44). Two studies found no significant results in relation to children’s healthy eating behavior (42, 43) and two studies found no significant associations with health behavior indicators (37, 38).

#### 4.3.2 Food to sooth

The use of sugar-rich foods for soothing was investigated in five studies (39, 41, 45–47). These include four longitudinal studies (39, 41, 45, 47) and one randomized controlled trial (46). The age range of the children studied varied from 6 months to 15 years, with a follow-up period ranging from 22 months to 11.5 years. Measurements were repeated two to four times within the specified age limits, and exposure was assessed solely through questionnaires. Five different exposure effects were considered (see Table 2).

**Table 2:** Exposure-outcome matrix of the associations between the use of sugar-rich food to sooth and prospective behavior.

FPP	Prospective child behavior	Prospective longitudinal studies				RCT
		Chong et al. (45)	Haszard et al. (39)	Jansen et al. (47)	Toh et al. (41)	Harris et al. (46)
Frequent use of food to soothe	Eating behavior					
	Food fussiness					
	Enjoyment of food					
	Emotional overeating			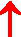		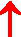
	Food responsiveness			•		
	Satiety responsiveness					
	Food reward				•	
	Health behavior indicator					
	Body fat mass	•		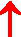		
	BMI	•	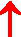	•^1^, 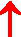 ^2^		

RCT, Randomized controlled trial; colors: green: healthy behavior, red: unhealthy behavior; 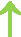/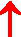: increased healthy/unhealthy behavior; 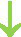/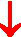: decreased healthy/unhealthy behavior; •: no significant association; ^1^Outcome at 3 and 4 years; ^2^Outcome at 6 and 10 years.

Two studies investigated the associations of frequent use to soothe with eating behavior (46, 47), and both concluded that the frequent use of sugar-rich food to soothe is significantly associated with emotional overeating. Food responsiveness was described by Jansen et al. (47) but did not show a significant increase in this behavior. Toh et al. (41) also found no significant association with food reward behavior.

The association with fat mass due to the use of sugar-rich food to soothe was examined in two studies: only one of the studies predicted an increase in fat mass because of this FPP (47), while the other study did not show any significant associations (45). Furthermore, three studies considered the effects on BMI (39, 45, 47). Two of these studies detected a significant increase in BMI at the ages of 5, 6, and 10 years as a consequence of exposure to sugar-rich foods trough this FPP (39, 47). However, this effect was not observed in the younger children (47). A third study found no such association (45). In summary, eight significant negative associations were found with the use of sugar-rich foods for soothing. Overall, two studies also found no significant associations between using food to sooth and healthy eating behavior in children (41, 47) and two studies also yielded no significant associations with health behavior indicators (45, 47).

#### 4.3.3 Food as a reward

The association between the use of sugar-rich foods as a reward and subsequent eating behavior was examined in four longitudinal studies (39, 41, 48, 49). The age of the participants ranged from 20 months to 11 years. The time frame of the follow-up measurement ranges from 22 months to 5 years of data collection later. The number of follow-ups varied from one to two times. Nine different types of subsequent behavior were evaluated (see Table 3).

**Table 3:** Exposure-outcome matrix of the associations between the use of food as a reward and prospective behavior.

FPP	Prospective child behavior	Prospective longitudinal studies			
		Flores-Barrantes et al. (49)	Haszard et al. (39)	Toh et al. (41)	Jansen et al. (48)
Frequent use of food as a reward	Food intake				
	Consumption				
	Energy-dense foods	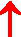			
	Nutrient-dense foods	•			
	Compliance with consumption recommendations for:				
	Fruit & Vegetables	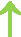 ^1^			
	Eating behavior				
	Food fussiness				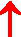 , •, ∀
	Enjoyment of food				
	Emotional overeating				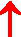 , ∀
	Food responsiveness				∀
	Satiety responsiveness				•, ∀
	Food reward			•	
	Health behavior indicator				
	BMI		•		

Colors: green: healthy behavior, red: unhealthy behavior; 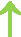/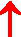: increased healthy/unhealthy behavior; 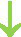/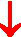: decreased healthy/unhealthy behavior; **•**: no significant association; ∀: association in the opposite direction (behavior as a predictor of increasing/decreasing use of parenting practice); ^1^measured a rare use of food as a reward: a rare use is associated with a higher adherence to the consumption recommendations.

Flores-Barrantes et al. (49) found a significant association with increased consumption of energy-dense and sugar-rich foods when sugar-rich foods were used as a reward, whereas no such significant associations were found for nutrient-dense foods as a reward. Furthermore, the results showed that low or decreasing use of sugar-rich foods as a reward favors compliance with fruit and vegetable intake recommendations in boys. Jansen et al. (48) reported significant increases in fussy eating and emotional overeating. In addition, they found that food fussiness, emotional overeating and food and satiety responsiveness in the eating behavior of the children were inversely predictive of the later use of the reward FPP, but this association was not significant for food fussiness and satiety responsiveness. Finally, Toh et al. (41) did not find significant associations with food reward, and Haszard et al. (39) found no significant associations with BMI. In summary, all four significant results indicated negative associations of using sugar-rich foods as a reward for children’s prospective behavior. However, one study also found non-significant associations of frequent use of food as a reward with children’s food intake (49), two studies found non-significant associations with healthy eating behavior in children (41, 48) and one study found no significant associations with health behavior indicators (39).

### 4.4 Associations with the FPP structure

The category “structure” includes the practices of monitoring unhealthy and sugar-rich foods, role modeling of healthy eating by parents and availability of energy-dense and sugar-rich foods.

#### 4.4.1 Monitoring

The associations of monitoring sugar-rich foods and prospective dietary behaviors were investigated in three studies (41, 43, 44). The children were aged 4–10 years, and follow-up assessments were conducted with a time interval of 1, 3, and 6 years. The exposure to sugar-rich foods and beverages was analyzed in relation to seven behavioral outcomes (see Table 4).

**Table 4:** Exposure-outcome matrix for associations between monitoring and prospective behavior.

FPP	Prospective child behavior	Prospective longitudinal studies		
		Barbosa et al. (44)	Derks et al. (43)	Toh et al. (41)
High level of monitoring	Food intake			
	Consumption			
	Energy-dense foods	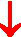		
	Nutrient-dense foods	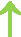		
	Eating behavior			
	Enjoyment of food		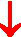	
	Emotional overeating		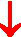	
	Food responsiveness		•	
	Satiety responsiveness		•	
	Food reward			•

Colors: green: healthy behavior, red: unhealthy behavior; 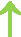/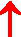: increased healthy/unhealthy behavior; 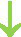/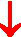: decreased healthy/unhealthy behavior; •: no significant association.

The results of a study by Barbosa et al. (44) showed that children whose parents exercised greater perceived monitoring at age 4 were less likely to follow energy-dense food patterns and were more likely to follow nutrient-dense food patterns at age 7. In addition, Derks et al. (43) indicated that high levels of sugar-rich food monitoring were associated with a decrease in food enjoyment and emotional overeating, while no significant associations were found for food and satiety responsiveness. An investigation conducted by Toh et al. (41) on food rewards also yielded no significant results. Overall, two studies indicated significant associations with a reduction in unhealthy behaviors due to the monitoring of sugar-rich foods and beverages (43, 44) and two studies found no significant associations with healthy eating behavior in children (41, 43).

#### 4.4.2 Parental healthy eating modeling

The modeling of healthy eating behaviors and low consumption of foods and beverages high in free sugars by parents was examined in the studies conducted by Flores-Barrantes et al. (49), Haszard et al. (39), and Toh et al. (41). The participants ranged in age from 20 months to 11 years, with follow-up assessments conducted once, and the time intervals between assessments varied from 22 months to 1 year. The outcomes investigated included subsequent food intake, eating behavior, and an indicator of health behavior (see Table 5).

**Table 5:** Exposure-outcome matrix on the associations between prior experience of healthy eating and prospective behavior.

FPP	Prospective child behavior	Prospective longitudinal studies		
		Flores-Barrantese et al. (49)	Haszard et al. (39)	Toh et al. (41)
Modeling of a healthy diet	Food intake			
	Consumption			
	Energy-dense foods	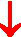		
	Nutrient-dense foods	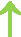		
	Compliance with consumption recommendations for:			
	Fruit & Vegetables	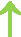		
	Water	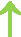		
	Eating behavior			
	Food reward			•
	Health behavior indicator			
	BMI		•	

Colors: green: healthy behavior, red: unhealthy behavior; 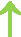/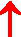: increased healthy/unhealthy behavior; 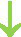/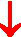: decreased healthy/unhealthy behavior; •: no significant association.

One study focused on the consumption of energy-dense and nutrient-dense foods and found that modeling healthy eating behavior by parents was associated with a reduction in unhealthy, sugar-rich food and beverage consumption and an increase in healthy food consumption (49). Additionally, children whose parents modeled healthy eating behavior and low consumption of sugar-rich foods and beverages were significantly more likely to adhere to recommendations for fruit, vegetable, and water intake. Conversely, the second study found no association with subsequent BMI (39), and the third study did not identify any relationship with food reward (41). Overall, one study demonstrated significant increases in healthy food intake in children, and indicated a reduction in unhealthy food intake because of the FPP type modeling. However, one study showed no significant associations between parental modeling of a healthy diet and healthy eating behavior in children (41) and another study showed no significant associations with health behavior indicators (39).

#### 4.4.3 Food availability (covert control)

The relationship between sugar-rich food availability and subsequent behavior in children was examined in five longitudinal studies (37, 41, 42, 49, 50). The participating children ranged in age from 18 months to 11 years, with follow-up assessments after 1 year (41) or 2 years (37, 42, 49, 50). The following exposures were examined: high availability of energy-dense/sugar-rich foods and beverages, high availability of nutrient-dense/low in free sugar foods and beverages, and low availability of energy-dense/sugar-rich foods and beverages (see Table 6).

**Table 6:** Exposure-outcome matrix of associations between food availability and prospective behavior.

FPP	Prospective child behavior	Prospective longitudinal studies				
		Boots et al. (42)	Boots et al. (37)	Fernando et al. (50)	Flores-Barrantes et al. (49)	Toh et al. (41)
High availability of energy-dense/unhealthy foods	Food intake					
	Consumption					
	Energy-dense foods				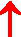	
	Nutrient-dense foods				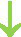 ^1^	
	Water				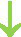	
	Energy density due to:					
	Food			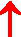		
	Food + dairy beverages			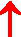		
	Food + all beverages			•		
High availability of nutrient-dense/healthy foods	Food intake					
	Consumption					
	Energy-dense foods				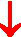	
	Nutrient-dense foods				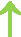 ^1^	
	Energy density due to:					
	Food			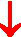		
	Food + dairy beverages			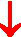		
	Food + all beverages			•		
	Eating behavior					
	Food reward					•
Low availability of energy-dense/unhealthy foods	Food intake					
	Consumption					
	Energy-dense foods/unhealthy snacks	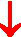			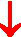	
	Nutrient-dense foods/healthy snacks	•				
	Compliance with consumption recommendations for:					
	Fruit & Vegetables				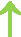	
	Water				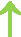	
	Food choice					
	Preference for:					
	Sweets		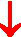			
	Fruit & Vegetables		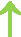			
	Health behavior indicator					
	BMI		•			

Colors: green: healthy behavior, red: unhealthy behavior; 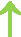/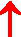: increased healthy/unhealthy behavior; 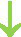/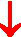: decreased healthy/unhealthy behavior; •: no significant association; ^1^This study classified 100 % fruit juice as nutrient dense.

The high availability of energy-dense sugar-rich foods revealed associations with increased consumption of such foods (49) and increased energy intake from food high in free sugar, as well as food plus dairy beverages (50). Moreover, the consumption of nutrient-dense foods and water has also decreased (49). Conversely, high availability of nutrient-dense foods was associated with a reduced intake of unhealthy foods and an increased intake of healthy foods (49). The low availability of energy-dense and sugar-rich foods exclusively resulted in positive behavioral outcomes. Two studies identified a reduction in the consumption of energy-dense foods (42, 49); however, no significant association was found with the consumption of nutrient-dense foods when these were not explicitly made more available (42). Adherence to recommendations for fruit, vegetable, and water intake was found to increase significantly when more healthy, low-sugar foods were available (49), as was preference for fruits and vegetables (37). In addition, the availability of healthy foods was significantly associated with a reduced preference for sugar-rich foods such as sweets over time (37).

Overall, two studies showed significant associations between the high availability of energy-dense foods and unhealthy food intake in children (49, 50), while one of these studies also showed non-significant associations in relation to energy density through the combination of food and all beverages (50). Two studies showed significant associations between the high availability of nutrient dense foods and healthy food intake in children (49, 50), while one of the studies found no significant correlations in relation to energy density through the combination of food and all beverages (50) and another study found no significant associations with healthy eating behavior in children (41). With regard to the low availability of energy dense foods, it can be summarized that two studies found significant associations in relation to the decrease in unhealthy food intake in children (42) as well as compliance with consumption recommendations of healthy foods (49), although one of these studies did not find any significant associations with the intake of nutrient-dense foods (42); another study showed significant results in relation to a healthier food choice preference of children, but no significant results in relation to health behavior indicators (37).

### 4.5 Associations with the FPP promotion of autonomy

The promotion of autonomy includes the encouragement of balance and variety, as well as nutrition education.

#### 4.5.1 Encouragement of balance and variety

Toh et al. (41) investigated the influence of promotion of balance and variety as a parenting practice on prospective food reward behavior, assessed through the willingness to work for a reward. The CFPQ scale was completed by one parent when the child was 5 years old, and the measurement of behavior was conducted after 1 year. The authors found that boys showed a significantly lower reward response to foods when their mothers promoted a balanced and varied diet, even with low amounts of energy-dense and sugar-rich foods. However, this result was not significantly demonstrated in girls (41).

#### 4.5.2 Nutrition education

The practice of nutrition education as a preventive measure against the intake of unhealthy sugar-rich foods and beverages was also examined only in the study by Toh et al. (41). Again, the CFPQ was used to assess the practice, and the focus of the investigation was on prospective food reward behavior. The authors reported that girls whose mothers provided nutritional knowledge regarding the health impacts of sugar-rich food and beverage consumption showed an increased willingness to work for a food reward.

### 4.6 Associations with the PFS permissiveness

Four studies examined permissiveness, or allowing sugar-rich foods and beverages (40, 41, 45, 49). The children ranged in age from 3.5 to 11.5 years, and prospective behavior was assessed at a minimum of 10 months (40) and a maximum of 11.5 years (45). Food intake, eating behavior, and indicators of health behavior were analyzed (see Table 7).

**Table 7:** Exposure-outcome matrix of the associations between permissiveness and prospective behavior.

PFS	Prospective child behavior	Prospective longitudinal studies			
		Chong et al. (45)	Flores-Barrantese et al. (49)	Liszewska et al. (40)	Toh et al. (41)
High permissiveness	Food intake				
	Consumption				
	Energy-dense foods		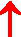		
	Nutrient-dense foods		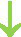		
	Water		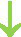		
	Eating behavior				
	Food reward				•
Low permissiveness/low child control	Food intake				
	Compliance with consumption recommendations for:				
	Fruit & Vegetables		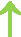		
	Health behavior indicator				
	Body fat mass	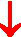			
	BMI	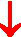		•, ∀	

Colors: green: healthy behavior, red: unhealthy behavior; 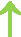/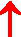: increased healthy/unhealthy behavior; 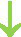/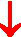: decreased healthy/unhealthy behavior; •: no significant association; ∀: association in the opposite direction (behavior as a predictor of increasing/decreasing use of parenting style).

Flores-Barrantes et al. (49) found a significant association between the high permissiveness of parents and increased consumption of energy-dense and sugar-rich foods by children. Consistent with this finding, they observed a reduction in the consumption of nutrient-dense foods and water. As a result of low permissiveness, adherence to recommendations for fruit and vegetable intake has increased. In contrast, Toh et al. (41) did not find a significant association between permissiveness and reward responsiveness to food, nor did another study find a significant association between permissiveness and BMI (40). However, a high BMI in children led parents to allow unhealthy, sugar-rich foods and beverages less frequently (40). Children whose parents practiced low permissiveness or did not allow control over food choice had significantly lower BMI and lower fat mass compared to those who were allowed to freely choose their food, including sugar-rich foods. Overall, one study found significant associations between high permissiveness and unhealthy food intake in children (49) and one study found a non-significant association with eating behavior in children (41). With regard to low permissiveness, one study showed significant associations with healthy food intake (49), one study found significant associations with more favorable health behavior indicators (45), while one study found no significant associations with health behavior indicators (40).

## 5 Discussion

This review aimed to systematically collect and narratively synthesize existing evidence from longitudinal studies to provide an overview of the associations of food parenting in relation to sugar-rich foods and beverages with prospective dietary behavior and health-related behavioral outcomes in children and adolescents. All 15 studies included in this review addressed the associations of parenting in relation to sugar-rich foods and beverages with prospective dietary behavior (food intake, food choices, and eating behavior) or indicators of health behavior (BMI and fat mass) in the target group. It was typical for a single study to analyze multiple FPP or PFS in relation to a specific outcome. Only four of the 15 studies focused exclusively on a single practice. Overall, eight different FPP and one PFS in relation to the handling of sugar-rich foods and beverages were identified.

### 5.1 Coercive control as not conducive to healthy dietary behavior

The investigations of practices of coercive control provide consistent results, suggesting that restriction of sugar-rich foods and beverages, using sugar-rich food to soothe or as a reward, contributes to unhealthy eating behaviors in children and adolescents (36, 37, 39–44, 46–49). Nine studies examined restrictive practices regarding sugar-rich foods and beverages, with six showing an increase in unhealthy behaviors or indicators and one showing a decrease in healthy behaviors. High restriction of sugar-rich foods resulted in higher consumption of energy-dense snacks and increased preference for sugar-rich foods, whereas the preference for fruits and vegetables decreased (37, 42). These outcomes are associated with an increased risk of being overweight (51). An increasing number of studies have already indicated a negative effect of high restriction of sugar-rich foods and beverages (52–54), which was confirmed by six of the nine longitudinal studies included in the synthesis of results related to FPP restriction.

Further results from the included studies provided evidence of long-term increases in emotional overeating and food responsiveness (43), as well as food rewards (41) based on high restriction of sugar-rich foods and beverages. These outcomes were positively associated with weight status (55) and demonstrated the long-term consequences of unhealthy eating behaviors resulting from FPP.

Regarding the use of sugar-rich food as a reward, other studies have indicated the development of negative behaviors such as increased consumption of energy-dense and sugar-rich foods and lower adherence to recommendations for fruit and vegetable intake (29, 49). Offering preferred unhealthy foods as rewards can influence children’s food preferences and consumption behavior, leading to an increased liking for rewarded unhealthy food and increased consumption (56, 57). Overall, all forms of coercive control in relation to exposure to sugar-rich foods and beverages were shown to have adverse effects and trigger the consumption of these foods. A possible explanation for these behaviors was provided by Birch et al. (56) and Newman and Taylor (58). They suggested that using a preferred food (like dessert) as a reward for eating another food (like broccoli) implies to the child that the rewarded food is more desirable, leading to a dislike or reduced enjoyment of the healthier option. This instrumental use of favorite or high-sugar foods can affect the child’s perception of the rewarded food, even if they had no prior preference or dislike for it. Additionally, Cooke (57) noted that frequent exposure to the reward food could increase its preference and consumption. However, our study focused on how parental exposure to high-sugar (unhealthy) foods affects consumption and did not explore the effects of using healthy foods as rewards. The use of healthy food as a reward may lead to other more health-promoting behaviors (44), which could be the subject of future research.

### 5.2 Structure as health-promoting approach

In the structure FPP category, practices such as monitoring sugar-rich foods and beverages, modeling healthy eating, and the availability of healthy and unhealthy, energy-dense foods were examined. These practices indirectly influence food intake through parental actions and home environment. Results suggest that structuring practices contribute to the development of healthy eating behaviors. Monitoring the consumption of sugar-rich foods and beverages showed a significant reduction in the enjoyment of food and emotional overeating (43). Consistent with this, studies have indicated that maternal monitoring leads to a decrease in sugar-rich eating behavior (59). Unlike controlling practices, structuring practices are assumed to support self-regulation (60), which is likely to explain these results. However, monitoring did not show associations with food responsiveness, satiety responsiveness, or food reward behavior (41, 43).

In relation to the modeling FPP type, decreased consumption of sugar-rich foods and increased intake of nutrient-dense foods, along with adherence to consumption recommendations was found (49). Thus, parental modeling of healthy food intake influences children’s behavior, with younger children being more influenced by parental modeling and older children being more influenced by their peers (53). Previous studies have shown increased consumption of healthy foods due to parental modeling (61–63). Modeling a healthy diet low in sugar-rich foods and beverages may help children understand the balance between healthy and unhealthy options. Moreover, the availability of energy-dense and sugar-rich foods in the home environment was associated with increased consumption, whereas the availability of nutrient-dense foods led to decreased consumption of unhealthy foods (49). In particular, the exclusive availability of healthy foods is crucial for positive effects. The results underline that parental modeling, and a healthy home food environment can be effective strategies to maintain the consumption of sugar-rich foods and beverages among children and adolescents at an acceptable level over the long term.

### 5.3 Autonomy as a gender-specific balancing act

Autonomy-promoting practices, like promoting balance and variety and providing nutrition education about sugar-rich foods, have shown mixed, gender-specific results in one longitudinal study (41). These practices significantly reduced food reward in boys but had no significant effect on girls. Conversely, nutrition education increased food reward behavior in girls but did not affect boys. This suggests that girls might be more influenced by societal and cultural pressures regarding weight control, leading to a greater willingness to work for food. Further longitudinal studies should examine how gender-specific combinations of these practices interact with socio-cultural factors to understand their impact on food reward behaviors.

### 5.4 Permissiveness—less is more

High parental permissiveness, characterized by allowing energy-dense and sugar-rich foods or beverages or relinquishing control over the child, has been associated with negative behaviors in children. High permissiveness is related to increased consumption of sugar-rich and nutrient-poor foods (49, 64). Conversely, low permissiveness was associated with greater compliance with fruit and vegetable consumption recommendations and better health indicators (45). Overall, the findings indicate, that granting children full control over food choices and portion sizes may adversely affect the consumption of sugar-rich foods and beverages. This highlights the importance of parental involvement in children’s nutrition to support the development of healthy behavioral patterns.

Assessing permissiveness and restrictive control reveals minimal differences. In the study by Flores-Barrantes et al. (49), permissiveness was measured with a single item, and low permissiveness effectively indicated overt restriction. While general restriction has been linked to the later development of unhealthy behaviors, low permissiveness seems to have a contrary effect. This discrepancy may arise from the bidirectional nature of parent–child interactions and the influence of various mediators leading to different outcomes (29). For example, how often a child requests unhealthy food and their availability at home are factors that remain unclear. Parents with low permissiveness might employ moderate restriction, which Jansen et al. (65) found to result in the lowest snack consumption.

### 5.5 Recommendations for practices of food parenting

Controlling practices such as the restriction of sugar-rich foods and beverages, the use of sugar-rich food to soothe, and as rewards exhibit long-term potential for the development of negative behaviors in children and adolescents. While they may serve short-term purposes, such as avoiding the consumption of unhealthy sugar-rich foods and beverages or encouraging the consumption of healthy foods through preferred food incentives, the assumption that sugar-rich foods as rewards can be sensible is not substantiated by long-term studies. Therefore, parents should refrain from using such approaches to deal with sugar-rich foods to promote the development of self-regulatory competencies in children. Even for overweight children, increasing coercive control in response to weight status appears to be counterproductive, highlighting the necessity of alternative non-food-based methods for soothing and rewarding.

In contrast, structuring practices can be advocated, as they indirectly influence children’s prospective behavior through role modeling, monitoring, and the availability of healthy foods. Parents can ensure the presence of nutritious foods in the home environment and demonstrate balanced dietary habits. This indirect and less overt approach can effectively limit the availability of unhealthy and sugar-rich foods and beverages while simultaneously fostering a preference for wholesome options, without imposing explicit prohibitions or restrictions that the child is consciously aware of (37). However, it is imperative for parents to possess a sound understanding of nutrition to distinguish foods that are healthy from those that are not. Identifying the added free sugars in unexpected food products can pose a challenge, as they may be listed under various alternative terms in ingredient lists. Additionally, parents should be cognizant of the recommendation for moderate consumption of natural sugars, such as those present in fruit juices or nectars (66).

Based on the results of the review, it can be assumed that combining structuring practices may enhance their positive effects compared with using a single practice. However, it is crucial not to combine them with highly controlling practices. Within the “structure” category, the practices of monitoring, and modeling healthy eating and the high and low availability of healthy and unhealthy foods were examined. These are indirect practices that do not represent communicated control over consumption, but attempt to promote or avoid the intake of certain foods through the individual’s behavior and the home environment (67). In contrast to the controlling practices, it is assumed that the structuring practices support the development of self-regulation, which has a fundamentally positive association with healthy eating behavior (60). Similarly, permissiveness in children’s diets should be avoided as overall both the very frequent allowance and severe restriction of unhealthy, sugar-rich foods and beverages were equally inappropriate.

With respect to autonomy-supportive practices, it is important to note that clear recommendations cannot be formulated owing to the limited available data. Nevertheless, the overarching aim of these practices is to facilitate and foster children’s capacity to independently make healthy dietary choices and to explore various options. Consequently, autonomy-supportive practices are not anticipated to induce adverse patterns in sugar-rich food consumption.

As shown in the results presented in Tables 1–3, 7, the review also highlighted that many of the relationships between children’s eating behaviors or health behavior indicators and food parenting practices are bidirectional: not only can parents influence the child’s eating behavior, but the child and respective health behavior indicators such as the BMI can also affect the food parenting practices and may lead to adjustments of parental practices (29, 68, 69). Models of parenting and child development typically assume a bidirectional relationship, and it is increasingly recognized that parent–child feeding models likely exhibit reciprocal dynamics as well (70). For instance, Berge et al. (71) report that in families with sibling dyads discordant in weight status, parents were more likely to use restrictive feeding practices with the overweight sibling, while applying pressure to eat and providing encouragement to eat with the healthy-weight sibling. However, our review reveals limited evidence to support a specific understanding of the potentially bidirectional nature of FPP. Existing longitudinal studies have primarily focused on the directional relationships between FPP and child eating behaviors across only two assessment points, with little research exploring how children’s eating behaviors (such as expression of hunger and satiety cues) or BMI might influence the application of a FPP (69). Future research should systematically investigate these bidirectional effects.

However, parents’ feeding practices and attitudes are significantly associated with children’s dietary habits and consumption (72, 73). Acting as gatekeepers, parents can restrict their child’s access to sugar (73). In summary, parental engagement in shaping children’s dietary habits plays a pivotal role in establishing healthy behaviors from early childhood. Parents should proactively adapt the home environment and modify their own behavior, thereby guiding their children toward healthy and well-balanced dietary behaviors through the mechanisms of observation and imitation.

### 5.6 Limitations and implications for further research

The study primarily focused on sugar-related parenting practices. However, given the complexity of isolating sugar-rich foods and beverages as a single factor of parenting practices in relation to food and nutrition as well as the lack of a clear concept for sugar-related parenting the review also encompasses studies on broader food-related parental practices and feeding styles (such as those that refer to parents’ handling of “unhealthy” or “energy-dense” foods in the parenting of their children). Therefore, the reported effects may not relate exclusively to sugar-rich foods and beverages (74). However, in parenting practices, individual critical foods, such as sugary items, often present exceptions that cannot be classified under typical parental practices. In future research it is therefore necessary to explicitly examine and investigate the handling of critical foods, such as sugar-rich foods and beverages, in the context of otherwise everyday parenting practices and their impact on the prospective dietary behavior of children and adolescents to derive reliable data and insights (7, 8).

The synthesis of this review only includes studies published between 2017 and 2023. Given the limited number of studies identified and included in this period, which was set for pragmatic reasons, we opted for a narrative synthesis to summarize the data. However, this approach precludes quantitative and statistical comparisons and analyses that could demonstrate significant correlations between FPP and PFS and their potential influence on prospective child behavior. Furthermore, this review highlights the scarcity of longitudinal research on sugar-related parenting practices. We recommend individualized surveys, standardized definitions and the inclusion of covariates such as BMI and socioeconomic status in future studies. Furthermore, we recommend using appropriate measures such as food preferences and the Healthy Eating Index to avoid relying solely on BMI, which could lead to bias. While a complete sugar ban is rare in parental practice, the emergence of “sugar-free parenting” is noteworthy (8) and warrants further exploration through prospective and observational studies. Overall, further research is needed to explore the broader outcomes as well as the social and cognitive dimensions of “sugar-related parenting” approaches. Sugar-free parenting, which involves raising kids without sugar, is not just one FPP or PFS, but rather a comprehensive approach to avoiding and eliminating sugar-rich and often even sugar-containing foods from the diets of both children and usually their parents. This method combines various practices, such as modeling, controlling the availability of such foods in the home environment, and restriction. Since our review yielded conflicting results for these practices in promoting a healthy diet, such an approach cannot be generally recommended based on current evidence. However, due to the interconnectedness of various practices in this approach, further longitudinal studies are needed to reliably determine its impact on children’s eating behavior.

## 6 Conclusion

In conclusion, this systematic review aimed to comprehensively gather and narratively synthesize existing evidence from longitudinal studies to provide a comprehensive overview of the associations of food parenting related to energy-dense, sugar-rich foods and beverages with various behavioral outcomes in children and adolescents. The goal was to extract actionable recommendations from these findings.

A comprehensive summary of empirical data was necessary to refine parent-targeted communication and intervention strategies for improving children’s dietary intake, promoting effective nutritional practices that beneficially influence children’s eating behaviors, and addressing parenting techniques that may unintentionally encourage the consumption of sugar-rich foods. Based on the results of this review, it can be deduced that practices involving the restriction of sugar-rich foods and beverages, as well as the use of such foods for rewards or soothing, are significantly associated with the development of negative behavioral outcomes and may subsequently contribute to overweight in children and adolescents. Conversely, our analysis underscores the vital role of a healthy home environment in positively shaping children’s prospective eating behaviors. Therefore, it is recommended that parents employ structuring practices, such as offering nutritious foods, closely monitoring dietary habits, and actively modeling balanced nutrition.

## Data Availability

The original contributions presented in the study are included in the article/Supplementary material, further inquiries can be directed to the corresponding author.
