# Supplementary material for: Associations of sugar-related food parenting practices and parental feeding styles with prospective dietary behavior of children and adolescents: a systematic review of the literature from 2017 to 2023
Source: Front Public Health. 2024 Aug 14;12:1382437. doi: 10.3389/fpubh.2024.1382437 (PMC11349743; doi:10.3389/fpubh.2024.1382437)
Supplement: Supplementary file 1 [file Data_Sheet_1.pdf]

Supplementary Material

1 Supplementary Data

The following is the Supplementary data to this article.

Tab. S1 Search strategy in the databases Web of Science and PubMed

Databases	Search strategy
Web of Science	(TS=(maternal* OR parent* OR "feed* style*" OR "parent* style*" OR "feed* practice*" OR "food* parent* practice*" OR "parent* practice*" OR "feed* strateg*" OR "parent* strateg*") AND TS=(sugar* OR sweet* OR "energy* dense*" OR carbohydrate* OR sucrose* OR glucose* OR unhealth*) AND TS=(behavio* OR habit* OR consequence* OR preference* OR intake* OR effect* OR consum* OR respons* OR result* OR choice*) AND LA=(English OR German) NOT TS=((*potato* OR *corn* OR maiz* OR animal* OR horse* OR mouse* OR mice* OR calf* OR cattle* OR drosophila* OR parenteral OR *iron* OR *yeast* OR escherichia* OR saccharum* OR sugarcane OR phenotyp* OR breed*)) NOT TI=((cell* OR bacteri* OR *plant* OR *sport* OR *cancer* OR diabet* OR disease* OR disorder* OR depression* OR hyperglyc* OR hypoglyc* OR breastfeed* OR milk* OR *syndrome* OR *caries* OR *pain* OR pregnan* OR cardio*))) AND (PY==("2023" OR "2022" OR "2021" OR "2020" OR "2019" OR "2018" OR "2017") AND DT==("ARTICLE")
PubMed	(((((("maternal*"[Title/Abstract] OR "parent*"[Title/Abstract] OR ("feed*"[Title/Abstract] AND "style*"[Title/Abstract]) OR ("parent*"[Title/Abstract] AND "style*"[Title/Abstract]) OR ("feed*"[Title/Abstract] AND "practice*"[Title/Abstract]) OR ("food*"[Title/Abstract] AND "parent*"[Title/Abstract] AND "practice*"[Title/Abstract]) OR ("parent*"[Title/Abstract] AND "practice*"[Title/Abstract]) OR ("feed*"[Title/Abstract] AND "strateg*"[Title/Abstract]) OR ("parent*"[Title/Abstract] AND "strateg*"[Title/Abstract])) AND ("sugar*"[Title/Abstract] OR "sweet*"[Title/Abstract] OR ("energy*"[Title/Abstract] AND "dense*"[Title/Abstract]) OR "carbohydrate*"[Title/Abstract] OR "sucrose*"[Title/Abstract] OR "glucose*"[Title/Abstract] OR "unhealth*"[Title/Abstract]) AND ("behavio*"[Title/Abstract] OR "habit*"[Title/Abstract] OR "consequence*"[Title/Abstract] OR "preference*"[Title/Abstract] OR "intake*"[Title/Abstract] OR "effect*"[Title/Abstract] OR "consum*"[Title/Abstract] OR "respons*"[Title/Abstract] OR "result*"[Title/Abstract] OR "choice*"[Title/Abstract])) NOT ("potato*"[Title/Abstract] OR "corn*"[Title/Abstract] OR "maiz*"[Title/Abstract] OR "animal*"[Title/Abstract] OR "horse*"[Title/Abstract] OR "mouse*"[Title/Abstract] OR "mice*"[Title/Abstract] OR "calf*"[Title/Abstract] OR "cattle*"[Title/Abstract] OR "drosophila*"[Title/Abstract] OR "parenteral"[Title/Abstract] OR "iron*"[Title/Abstract] OR "yeast*"[Title/Abstract] OR "escherichia*"[Title/Abstract] OR "saccharum*"[Title/Abstract] OR "sugarcane"[Title/Abstract] OR "phenotyp*"[Title/Abstract] OR "breed*"[Title/Abstract])) NOT ("cell*"[Title] OR "bacteri*"[Title] OR "plant*"[Title] OR "sport*"[Title] OR "cancer*"[Title] OR "diabet*"[Title] OR "disease*"[Title] OR "disorder*"[Title] OR "depression*"[Title] OR "hyperglyc*"[Title] OR "hypoglyc*"[Title] OR "breastfeed*"[Title] OR "milk*"[Title] OR "syndrome*"[Title] OR "caries*"[Title] OR "pain*"[Title] OR "pregnan*"[Title] OR "cardio*"[Title])) AND 2017/01/01:2023/12/31[Date - Publication] AND "journal article"[Publication Type] AND ("english"[Language] OR "german"[Language])

Fig. S1 Construct of Food Parenting Practices (FPP) (Musher-Eizenman et al., 2019)

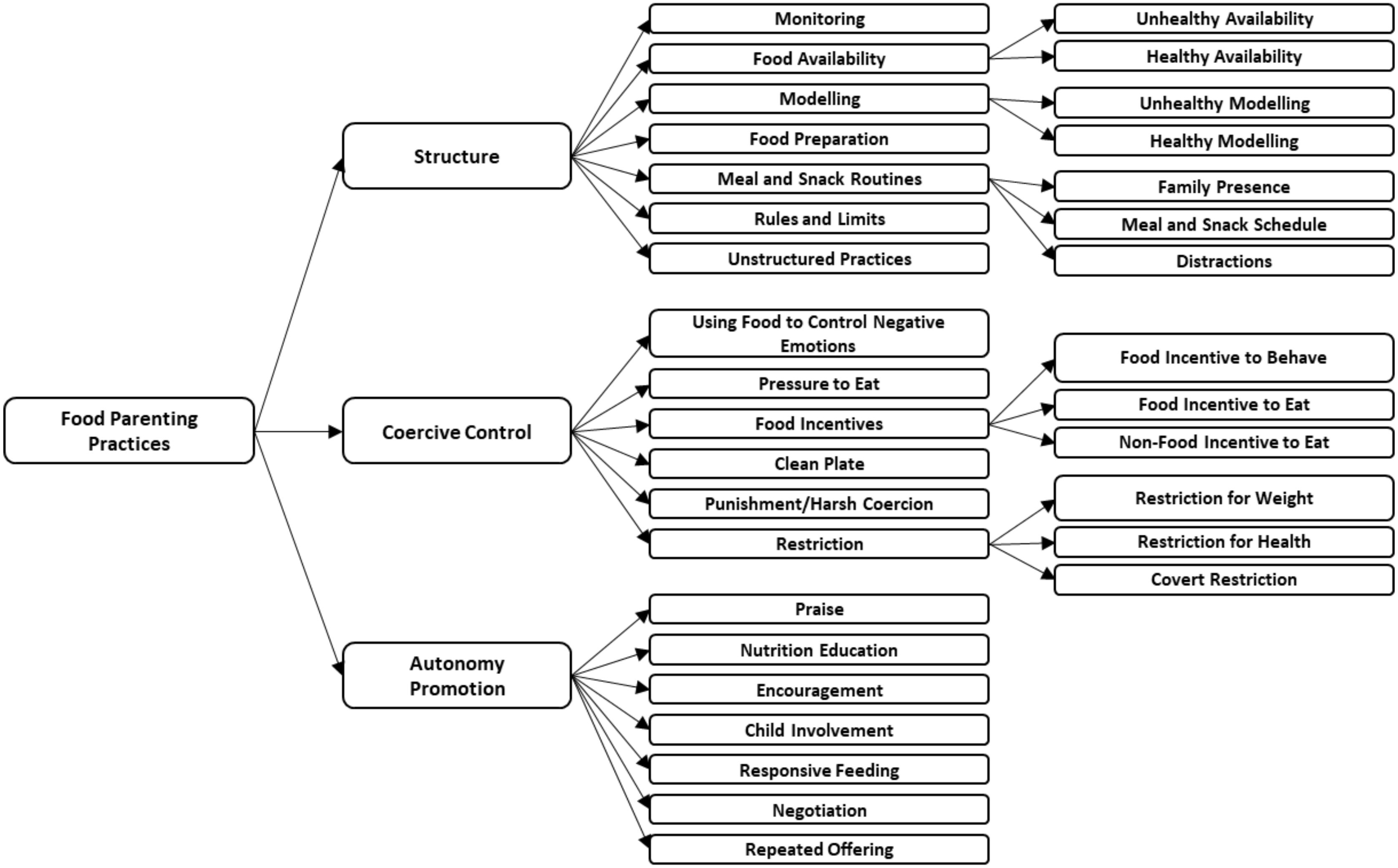

Fig. S2 Construct of Parental Feeding Styles (PFS) (Shloim et al., 2015)

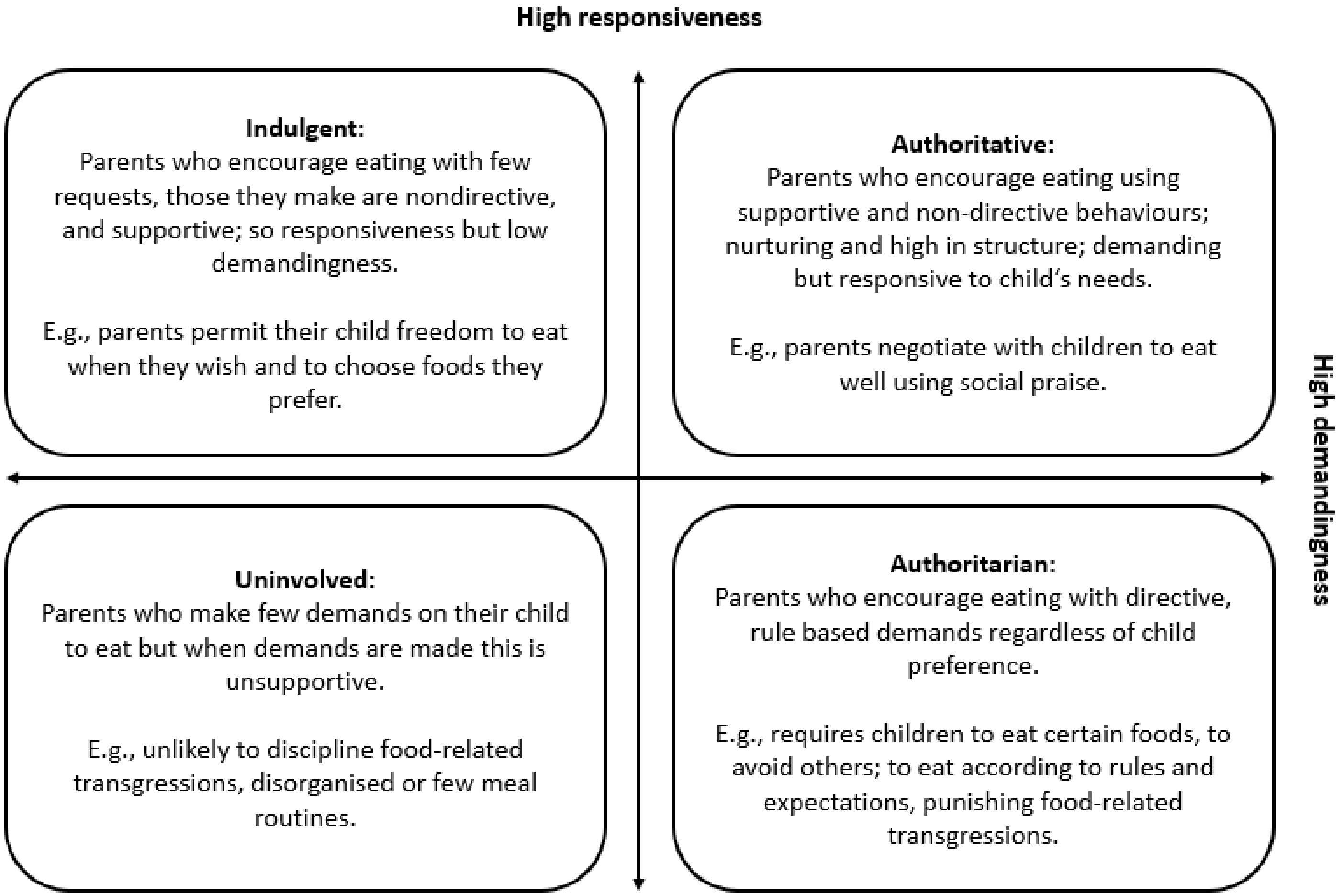

Tab. S2 Overview of studies reviewed

Prospective longitudinal studies						
**#**	**Reference**	**Journal**	**Sample sizes / child age**	**Frequency of Follow-Up (Duration)**	**Measuring method of food parenting practices**	**Measuring method of child behavior or health indicator**
1	BARBOSA et al. 2023 (Portugal)	Journal of Human Nutrition and Dietetics	n = 3,272 children and their mothers / 0-10 years	3 (4, 3, 3 years)	CFQ (Birch et al. 2001): Perceived responsibility, perceived parent weight, perceived child weight, concern about weight, restriction, pressure to eat, monitoring; (Ogden et al. 2006): Overt and Covert control-scale	FFQ: child's diet; Measurement of height and weight for zBMI determination
2	BOOTS et al. 2018a (Australia)	Appetite	n = 252 children (127 boys, 125 girls) and their mothers / 3-6 years	1 (3 years)	CFQ (Birch et al. 2001): Restriction; (Ogden et al. 2006): Covert control-scale	Adapted FFQ of the Anti-Cancer-Council Virginia (Files & Ireland 1996): Snack consumption
3	BOOTS et al. 2019 (Australia)	Appetite	n = 106 children (49 boys, 57 girls) and their mothers / 4-6 years	1 (2 years)	CFQ (Birch et al. 2001): Restriction; (Ogden et al. 2006): Covert control-scale	Face-to-Face interview: Food preference assessment by photo rating
4	CHONG et al. 2017 (UK)	Appetite	n = 7,312 children and their mothers / 3.5-15 years	2 (3.5; 11.5 years)	1-item: Parental control over food choice, food to soothe	Measurement of height and weight for zBMI determination, measurement of fat mass by DEXA
5	DERKS et al. 2017 (Netherlands)	American Journal of clinical nutrition	n = 4,689 children (49.5 % boys) and their mothers / 4-10 years	2 (2; 4 years)	CFQ (Birch et al. 2001): (Adapted) restriction-scale, adapted scale of concern about weight	Measurement of height and weight for zBMI determination, measurement of fat mass and fat-free mass by DEXA
6	DERKS et al. 2019 (Netherlands)	Appetite	n = 3,514 children (50.9 % girls) and their mothers / 4-10 years	1 (6 years)	CFQ (Birch et al. 2001): Restriction, monitoring	CEBQ (Wardle et al. 2001): Food responsiveness, enjoyment of food, emotional overeating, satiety responsiveness
7	FARROW et al. 2018 (UK)	International Journal of Environmental Research and Public Health	n = 39 children and their mothers / 3-7 years	1 (2 years)	Observation of verbal and physical restriction at lunch	Measurement of height and weight for zBMI determination
8	FERNANDO et al. 2018 (Australia)	Nutrients	n = 209 children (103 boys, 106 girls) and their mothers / 1.5-3.5 years	1 (2 years)	7-items (adapted from MacFarlane et al. 2010): Availability of fruits, vegetables, n*on-core* snacks and n*on-core* beverages	24-h dietary recall: Measurement of dietary energy density
9	FLORES-BARRANTES et al. 2021 (6 European countries)	Nutrients	n = 2.967 children (50.4 % girls) and one parent (93.5 % mothers) / 6-11 years	1 (2 years)	9-items: Availability of nutrient-dense and energy-dense food; 1-item: Food as a reward, modeling of fruit intake and permissiveness	9-items: Consumption frequency of nutrient-dense and energy-dense food
10	HASZARD et al. 2019 (Australia & New Zealand)	Appetite	n = 723 children and their mothers / 1.66-5 years	2 (1.83; 3.33 years)	CFPQ (Musher-Eizenman et al. 2007): Food as a reward, modeling, restriction for health, emotional regulation	Measurement of height and weight for zBMI determination
11	JANSEN et al. 2019 (Netherlands)	Journal of Nutrition	n = 3.960 children (49.3 % boys) and one parent (89 % mothers) / 0.5-10 years	4 (2.5, 3.5, 5.5, 9.5 years)	1-item: Food to soothe	CEBQ (Wardle et al. 2001): Food responsiveness, emotional overeating; Measurement of height and weight for zBMI determination, measurement of fat mass and fat-free mass by DEXA

12	JANSEN<br>et al. 2020<br>(Netherlands)	Pediatric Obesity	n = 3.642 children (50.7 % girls) and parents<br>4-9 years	1 (5 years)	CFQ (Birch et al. 2001):<br>Restriction scale (2 items for food as reward)	CEBQ (Wardle et al. 2001):<br>Food responsiveness, emotional overeating, satiety responsiveness<br>Food Fussiness scale of the CEBQ (4 years)<br>Picky Eating scale of CEBQ (9 years)<br>Measurement of height and weight for zBMI determination
13	LISZEWSKA<br>et al. 2018<br>(Poland)	Appetite	n = 526 children (43.2 % boys) and one parent (91.6 % mothers) /<br>6-11 years	1 (0.83 years)	CFPQ (Musher-Eizenman et al. 2007):<br>Restriction for health and for weight control;<br>2-items: Permission for unhealthy food	Measurement of height and weight for zBMI determination
14	TOH<br>et al. 2021<br>(Singapore)	PLOS ONE	n = 430 children and their mothers /<br>5-6 years	1 (1 year)	CFPQ (Musher-Eizenman et al. 2007):<br>Restriction for health and for weight control, child control, emotional regulation, food as a reward, encourage balance/variety, teaching nutrition, healthy environment, modeling, and monitoring	*Food Reward Task*:<br>Implementation of a write-for-food task
Randomized controlled trial						
#	Reference	Journal	Sample sizes /<br>child age /<br>Intervention	Frequency of Follow-Up (Duration)	Measuring method of food parenting practices	Measuring method of child behavior or health indicator
15	HARRIS<br>et al. 2020<br>(USA)	Pediatric Obesity	n = 207<br>mother-child dyads / 3 weeks-2 years / 7 appointments<br>IG: n = 105 dyads; Responsive Parenting<br>KG: n = 102 dyads; Home safety	1 (1.83 years)	BBNQ (Stifter et al. 2011):<br>Food to soothe	CEBQ (Wardle et al. 2001):<br>Emotional overeating
